# A Promising Future of Ferroptosis in Tumor Therapy

**DOI:** 10.3389/fcell.2021.629150

**Published:** 2021-06-09

**Authors:** Hui Wang, Danfeng Lin, Qianqian Yu, Zhouqi Li, Cameron Lenahan, Ying Dong, Qichun Wei, Anwen Shao

**Affiliations:** ^1^Department of Medical Oncology, Key Laboratory of Cancer Prevention and Intervention, Ministry of Education, The Second Affiliated Hospital, Zhejiang University School of Medicine, Hangzhou, China; ^2^Department of Breast Surgery, Key Laboratory of Cancer Prevention and Intervention, Ministry of Education, The Second Affiliated Hospital, Zhejiang University School of Medicine, Hangzhou, China; ^3^Department of Radiation Oncology, Key Laboratory of Cancer Prevention and Intervention, Ministry of Education, The Second Affiliated Hospital, Zhejiang University School of Medicine, Hangzhou, China; ^4^Burrell College of Osteopathic Medicine, Las Cruces, NM, United States; ^5^Center for Neuroscience Research, Loma Linda University School of Medicine, Loma Linda, CA, United States; ^6^Department of Neurosurgery, The Second Affiliated Hospital, Zhejiang University School of Medicine, Hangzhou, China

**Keywords:** ferroptosis, iron, glutathione, anti-tumor therapy, nanoparticle, radiotherapy

## Abstract

Currently, mechanisms and therapeutic approaches have been thoroughly studied in various prevalent malignant tumors, such as breast and lung cancer. However, there is inevitable tumor progression and drug resistance. Uncovering novel treatment strategies to inhibit tumor development is important. Ferroptosis, a form of cell death associated with iron and lipid peroxidation, has drawn extensive attention. In this paper, we reviewed the underlying mechanisms of ferroptosis (i.e., iron, glutathione, and lipid metabolism) and its role in various tumors (i.e., lung cancer, liver carcinoma, breast cancer, and pancreatic cancer). Moreover, we summarized ferroptosis-related anti-tumor drugs and emphasized the potential of combined treatment of anti-tumor drugs and radiotherapy in an effort to provide novel anti-tumor treatments.

## Introduction

The last two decades have witnessed a decrease in cancer mortality due to rapid developments of tumor diagnosis and comprehensive therapies (i.e., surgery, chemotherapy, radiotherapy, targeted therapy, and immunotherapy). However, the recurrence and metastasis rates of some tumors remain high. For example, the recurrence of non-small cell lung cancer (NSCLC) in the early and advanced stages are approximately 20 and 50%, respectively (Lou et al., 2014). The 5-year survival rate of pancreatic cancer is 5%(Chen et al., 2016). Therefore, it is imperative that we find new treatment strategies. Recent studies have shown an important role of ferroptosis in various diseases, such as tumors (Gout et al., 2003; Yang et al., 2014; Greenshields et al., 2017), acute kidney failure (Skouta et al., 2014), liver injury (Angeli et al., 2014; Friedmann Angeli et al., 2014), and heart injury (Gao et al., 2015).

Ferroptosis is a unique form of cell death characterized by excessive accumulation of lipid hydroperoxides to lethal levels in an iron-dependent pathway (Stockwell et al., 2017). Chen et al. (2021) indicated that ferroptosis played a dual role in tumor progression. On one hand, some drugs can induce ferroptosis to suppress tumor growth by causing cysteine depletion or by inactivating glutathione peroxidase (Guo et al., 2018). On the other hand, ferroptosis could evoke immunosuppression to accelerate tumor growth by triggering a cellular inflammatory response (Murao et al., 2021). Combining ferroptosis inhibitors with immunotherapy may be a novel strategy for tumor therapy.

Regarding the underlying mechanisms, extensive studies have suggested that ferroptosis can be regulated by many inhibitors and inducers (Liang et al., 2019). Ferroptosis is reportedly inhibited by lipid hydroperoxide inhibitors and iron chelators, such as deferoxamine (DFX) and desferrioxamine mesilate. Additionally, ferroptosis can be pharmacologically triggered (Liang et al., 2019) by system Xc- inhibitors, by inactivation of glutathione peroxidase 4 (GPX4) via experimental chemical compounds [e.g., Ras-selective lethal small molecule 3 (RLS3) and erastin], and by various drugs [e.g., sulfasalazine (SAS), artemisinin, and sorafenib (Zhu et al., 2020)]. Emerging evidence also illustrates a potential role of ferroptosis-related therapies in tumor inhibition. A study investigating 114 tumor cell lines revealed that diffuse B cell lymphomas and malignant renal tumor cells were extremely vulnerable to erastin (Yang et al., 2014), an agent that could inhibit the growth of tumor cells with a rat sarcoma (RAS)-mutated viral oncogene (Yang and Stockwell, 2008). Sato et al. (2018) also supported the role of erastin by showing that short erastin pretreatment was sufficient to synergize with cisplatin to kill tumor cell death. Similarly, Lei et al. (2020) demonstrated that ferroptosis inducers endowed radio-resistant tumor cells with radiation sensitivity via inactivation of SLC7A11 (a protein controlling the transport of cystine and glutamate), leading to ferroptotic cell death. However, the response to ferroptosis regulators, as well as the sensitivity to ferroptosis, varied among malignant tumors. Herein, we summarized the mechanisms of ferroptosis in tumors, and paid attention to ferroptosis-related anti-tumor therapies.

## Mechanisms of Ferroptosis

### The Role of Iron and Iron Chelators

Although iron is significant for cell survival (Ray et al., 2012), iron overload can disrupt the balance of iron metabolism, thus damaging cells by increasing reactive oxygen species (ROS) accumulation and by triggering ferroptosis (Gkouvatsos et al., 2012). There are many iron-related proteins involved in maintaining iron homeostasis. Ferritin can safely store mineralized redox-inactive iron, and transferrin can specifically bind to iron-transferrin receptor 1 (TfR1) at the cell surface to mediate cellular iron intake (Gao et al., 2015). During the process of iron distribution, the initial step is iron (Fe^3+^) importation via TfR1. In the presence of the metalloreductase, six-transmembrane epithelial antigen of the prostate 3 (STEAP3), within the endosome, Fe^3+^ is reduced to ferrous iron (Fe^2+^), which is released into the cytoplasm by the divalent metal transporter 1. Subsequently, ferrous iron is either stored in ferritin or exists as free Fe^2+^ in the cytoplasm. In the last step, Fe^2+^ is exported out of cells through the membrane protein, ferroportin, before being oxidized to Fe^3+^. When there is excess Fe^2+^ due to excess iron intake, reduced ferritin, and decreased export activity, it can induce ferroptosis through the Fenton reaction and the lipoxygenase pathway to cause cell death. Compared with ferroptosis-resistant cells, Ras-mutation ferroptosis-sensitive tumor cells had a higher expression of TfR1 and a lower expression of ferritin (Brown and Mercurio, 2020), suggesting that iron overload could trigger ferroptosis in cancer cells (Dixon and Stockwell, 2014). Other research to support the role of iron imbalance in tumors showed that iron accumulation may increase the risk of breast tumors by disrupting iron homeostasis (Diallo et al., 2016). Dixon et al. (2012) showed that DFX can facilitate malignant cell destruction by increasing iron-induced ROS. Lui et al. (2015) also found that DFX served as an anti-tumor drug to inhibit malignant cell proliferation. Paubelle et al. (2013) also reported anti-leukemia activity of DFX, and observed a recurrent acute myelogenous leukemia patient obtaining complete remission.

**FIGURE 1:**
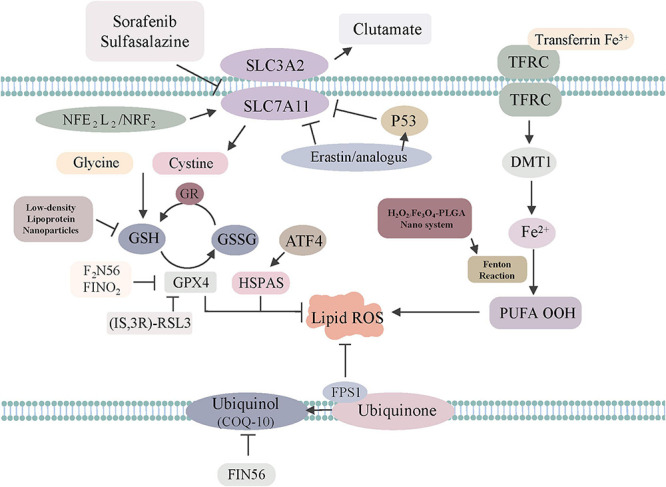
The regulatory mechanisms of ferroptosis-related drugs or genes. Sorafenib, sulfasalazine, erastin/analogs and p53 inhibit SLC7A11 to trigger ferroptosis. GPX4 is directly inhibited by (1S, 3R)-RSL3, F2N56, FINO2, HSPAS. NADPH–FSP1–CoQ10 ferroptosis surveillance pathway acts in parallel to the GPX4 pathway. Low-density, lipoprotein, Nanoparticles can regulate GSH to induce ferroptosis. H_2_O_2_/Fe_3_O_4_-PLGA nanosystem can regulate Fenton reaction to induce ferroptosis.

To summarize, the excess absorption of iron contributes to elevated risks of tumors via iron-related lipid peroxidation, while the accumulation of iron can trigger ferroptosis to induce tumor cell death (Figure 1). Further studies are warranted and necessary to reveal the explicit role of tumor-related ferroptosis, which may provide a rational application of ferroptosis-related therapies.

### The Role of Glutathione Peroxidase 4 and System Xc-

The GPX4-glutathione (GSH)-cysteine axis and GPX4-associated lipid peroxidation are important for triggering ferroptosis (Yang et al., 2014). GPX4 can reduce hydroperoxides to block the lipoxygenase pathway to inhibit ferroptosis. Inactivating GPX4 with RSL3 (Sui et al., 2018), ML162 (Moosmayer et al., 2021), and ML210 (You et al., 2021) could increase the production of lipid ROS, thereby inducing ferroptosis. Friedmann Angeli et al. (2014) observed that GPX4 knockout contributed to renal failure in mice, but this injury could be rescued by ferrostatin-1, an agent that could decrease the level of iron to inhibit iron-dependent lipid peroxidation. Regarding other regulators affecting the GPX4-GSH-cysteine axis, one inhibitor is erastin, which functions by reducing the production of GSH and oxidation of nicotinamide adenine dinucleotide phosphate and lysophosphatidylcholines (Yang et al., 2014). Conversely, increasing the GSH levels reversed the lethal effect of erastin in U2OS cells (Ho et al., 2007). As for the third regulator, RSL3, it can directly target GPX4 and generate ROS without GSH depletion (Sui et al., 2018). Regarding the role of GPX4 in tumors, Zhang et al. (2020) detected that inhibition of GPX4 with RSL3 could enhance the anti-tumor effect of cisplatin. Other research showed that GPX4 inactivation could increase the vulnerability of renal clear-cell carcinomas ferroptosis by increasing lipid peroxidation. Recently, Ubellacker et al. (2020) found that melanoma did not easily disperse through the vasculature as a result of tumor cell death caused by GPX4 inhibitor-dependent ferroptosis. This cancer tends to metastasize through the lymphatic system since the microenvironment of lymph could protect melanoma cells from ferroptosis. In short, GPX4 represents a potential target in triggering cancer cell death (Figure 1).

System Xc-, the upstream node of the GPX4–GSH–cysteine axis, is a crucial factor for ferroptosis (Chen L. et al., 2020; Xu et al., 2020). System Xc- locates on the cell plasma membrane, and it participates in exchanging extracellular cystine for intracellular glutamate to maintain redox homeostasis, followed by the reduction of cysteine and syncretization of GSH. Ferroptosis can be triggered by system Xc- inhibition via SAS in HT1080 cells. However, increased cystine uptake can inhibit this process. Furthermore, as system Xc- is composed of SLC3A2 and xCT, modulating xCT can influence ferroptosis (Dixon et al., 2012). It has been shown that reducing the expression of xCT by specific RNAi fostered the ferroptosis of tumor cells and increased the anti-cancer effect of erastin. Increased expression of xCT reduced erastin-triggered ferroptosis (Dixon et al., 2012). Sehm et al. (2016) found that gliomas with increased expression of xCT were associated with higher vulnerability to co-treatment with temozolomide-erastin. Therefore, investigation of system Xc- may have potential in tumor therapy.

### Other Pathways Related to Ferroptosis

Ferroptosis can also be directly inhibited by ferroptosis suppressor protein 1 (Bersuker et al., 2019). This protein could reduce coenzyme Q10 to block lipid peroxidation (Zhang et al., 2020). The new NADPH–FSP1–CoQ10 pathway functions in parallel to the canonical glutathione-based GPX4 pathway (Zhang et al., 2020). FSP1 inhibitors (Doll et al., 2019) may be a novel way to reduce ferroptosis resistance in tumors. Furthermore, Song et al. (2018) found that activating AMP-activated protein kinase (AMPK) in the AMPK-SREBP1 pathway could enhance the transcription of BECN1 (Wang et al., 2020), an enzyme responsible for increasing intracellular glutamicum level, thus preventing cells from undergoing ferroptosis.

## Ferroptosis-Associated Transcription Factors in Tumors

To date, several transcription factors, such as p53 and nuclear factor erythroid-2 related factor 2 (NRF2), have been found to play significant roles in ferroptosis (Figure 1).

### p53

The p53 is a tumor suppressor gene that is important in regulating the growth, differentiation, and death of cells. Recently, p53 has been proven to have a bidirectional effect in ferroptosis (Jiang et al., 2015a; Kang et al., 2019). On one side, p53 can promote ferroptosis by decreasing the expression of xCT in tumors (Jiang et al., 2015a; Huang et al., 2018; Kang et al., 2019; Hong et al., 2021). Additionally, p53 can also trigger ferroptosis in MCF7 (human breast cancer) and human osteosarcoma U2OS (human osteosarcoma) by suppressing xCT expression, leading to cell death (Jiang et al., 2015b). Moreover, p53 upregulated the expression of spermine N1-acetyltransferase 1 (SAT1) and glutaminase 2, contributing to ferroptosis. Specifically, SAT1 (Abdelmonem and Ohno, 1978) has been proven to be a transcriptional target of p53 in H1299, MCF7, U2OS, and A375 cells (Ou et al., 2016). The depletion of SAT1 suppressed p53(3KR) and p53-related ferroptosis, suggesting that SAT1 expression may be beneficial in cancer therapy (Ou et al., 2016). Glutaminase 2, another target of p53, was also involved in ferroptosis via glutaminolysis in tumor cells (Hu et al., 2010).

Conversely, p53 suppressed ferroptosis by limiting the expression of dipeptidyl peptidase 4 (DPP4) (Xie et al., 2017) or by inducing the activity of cyclin-dependent kinase inhibitor 1A (CDKN1A/p21) (Tarangelo et al., 2018). Xie et al. (2017) revealed that upregulation of DPP4 could inhibit ferroptosis in SW48 and HCT116 cells. The loss of p53 decreased the production of DPP4, and promoted the formation of the DPP4-NADPH oxidase 1 complex, finally contributing to reducing lipid peroxidation. Inactivating DPP4 and blocking the formation of the DPP4-tumor protein 53 (TP53) complex can limit erastin-induced ferroptosis (Xie et al., 2017). Moreover, wild-type p53 could increase the intracellular level of GSH to reduce the production of lipid-ROS by targeting p21, thus protecting cells from ferroptosis.

### NRF2

NRF2 is a common leucine zipper transcription factor (Ma, 2013). It is essential for redox homeostasis, and it works by combining with antioxidant response element oxidation genes. Under normal conditions, the Kelch-like ECH-related protein 1 (KEAP1)-NRF2 complex exists in the plasma membrane. NRF2 transports to the nucleus to induce the transcription of ARE-dependent target genes when under stress (Jamil et al., 2020). NRF2 regulates the metabolism of multiple cancer cells (Zimta et al., 2019), and protects malignant cells from oxidative damage. When activated by tumor cells, NRF2 assists in maintaining tumor cell redox homeostasis to protect malignant cells from anti-tumor drugs (Xiang et al., 2014).

Thus far, many studies have assessed the link between NRF2 activation and ferroptosis resistance. Bai et al. (2019) confirmed that activating the NRF2 pathway suppressed ferroptosis in a hepatocellular carcinoma (HCC) mouse model. Erastin and sorafenib can trigger ferroptosis by inactivating NRF2–KRAP1 complex. Recently, a study carried out by Roh et al. (2017) showed that NRF2 inhibition increased sensitivity of both head and neck cancer to ferroptosis. A study showed that suppressing the SQSTM1–KEAP1–NRF2 pathway facilitated ferroptosis in HCC cells with co-treatment of erastin and sorafenib (Inami et al., 2011). Overall, NRF2 inhibition may be a promising therapy for tumors.

## Ferroptosis in Relevant Malignant Tumors

Despite comprehensive therapies, it is difficult to halt the progression of tumors with a high recurrence or mortality rate (i.e., lung cancer, HCC, malignant breast tumors, and pancreatic cancer). A better understanding of ferroptosis-related metabolism in malignancy (Table 1) may contribute to finding novel anti-tumor therapies.

**TABLE 1:** Overview of pathways and functions of ferroptosis-associated agents and proteins.

**Types of cancer**	**Agents/proteins**	**Pathway**	**Function**
**Lung cancer**	DFX (Sarno et al., 2018)	Reducing the intracellular iron level	Anti-tumor effects
	Erastin and analogs (Li Y. et al., 2020)	Inhibiting the NRF2/xCT/	Enhance the effect of cisplatin and resensitize chemoresistance
	Erastin and APAP (Hammad et al., 2019)	Inhibit the Xc/NRF2/HO-1	Anti-tumor effects
	Erianin (Chen P. et al., 2020)	in a calcium/calmodulin-related way to trigger ferroptosis	Anti-tumor effects
	Erastin/sorafenib (Li Y. et al., 2020)	Inhibit the xCT	Anti-tumor effects
	Dihydroartemisinin (Yuan et al., 2020)	Inhibit slc7a11	Anti-tumor effects
	Sulforaphane (Iida et al., 2021)	Inhibit slc7a11	Anti-tumor effects
	Zinc (Palmer et al., 2019)	Intoxication to trigger ferroptosis	Anti-tumor effects
	Celastrol/Erastin (Liu et al., 2021)	Increasing ROS/mitochondrial Oxidation	Anti-tumor effects
	Long non-coding RNA NEAT1 (Wu and Liu, 2021)	Decreased the levels of ACSL4, SLC7A11, and GPX4	Anti-tumor effects
**HCC**	Sorafenib (Louandre et al., 2013)	Inhibiting SCL7A11	Anti-tumor effects
	Solasonine (Jin et al., 2020)	Inhibit GPX4	Anti-tumor effects
	ARF2 (Sun et al., 2016)	Inhibit p62-Keap1-NRF2 pathway to trigger ferroptosis	Anti-tumor effects
	DAZAP1 (Wang et al., 2021)	Inhibit system Xc^–^	Anti-tumor effects
	Activated AMPK/SREBP1 pathway (Wang et al., 2021)	inhibit the transcription of BCAT2 to reduce glutamate to trigger ferroptosis	Anti-tumor effects
	QSOX1 (Sun et al., 2021)	Inhibition of NRF2 to promote sorafenib-induced ferroptosis	Anti-tumor effects
	OS-related Sigma 1 receptor (S1R) (Bai et al., 2017)	Inhibit ferroptosis	Cause sorafenib resistance
**Breast cancer**	Lapatinib (Ma et al., 2017)	elevating the level of ROS	Anti-tumor effects
	Neratinib (Nagpal et al., 2019)	elevating the level of ROS	Anti-tumor effects
	Sulfasalazine (SAS) (Yu H.C. et al., 2019)	Inhibit System xc-	Anti-tumor effects
	Siramesine (Villalpando-Rodriguez et al., 2019)	elevating the level of ROS and lipid peroxidation	Anti-tumor effects
	GSK3 β/NRF2 (Wu et al., 2020)	Enhance the effect of erastin-induced ferroptosis	Anti-tumor effects
	erastin@FA-Exo (Limoni et al., 2019)	promoting depletion of glutathione and excess production of ROS	Anti-tumor effects
	Curcumin (Li R.H. et al., 2020)	elevating the level of ROS and lipid peroxidation	Anti-tumor effects
**Pancreatic cancer**	Non-oxidative dopamine, Baicalein (Donald et al., 2012)	elevating the level of ROS and iron	Anti-tumor effects
	Artesunate with GRP78 inhibition (Song et al., 2020)	elevating the level of ROS and iron	Anti-tumor effects
	Ruscogenin (Yamaguchi et al., 2018)	Regulating transferrin and ferroportin	Anti-tumor effects
	Piperlongumine, Cotylenin A, and sulfasalazine (Badgley et al., 2020)	elevating the level of ROS and iron	Anti-tumor effects
	ADP Ribosylation Factor 6 (ARF6) (Hou et al., 2016)	RSL3-induced lipid peroxidation	Regulate gemcitabine resistance to present anti-tumor effects
	HSPA5 (Zhang et al., 2019)	Inhibit GPX4	Anti-tumor effects
	Artesunate (Wang et al., 2019)	elevating the level of ROS and iron	Anti-tumor effects

### Lung Cancer

Lung cancer and metastases are major concerns worldwide (Kuang and Wang, 2019). The recommended therapies for lung cancer include surgery, conventional chemotherapy (Travert et al., 2020), targeted therapy, immunotherapy (Koleczko and Wolf, 2020), and radiotherapy (Sun et al., 2020). Within the treatment process, systemic toxicity (Bourzac, 2014), drug resistance, and immune evasion (Liu et al., 2020) seem to be inevitable. In this regard, nanoparticle formulation for lung cancer treatment is emerging (Barenholz, 2012; Chiang et al., 2020), but the efficacy remains unknown (Chen Y. et al., 2020). Drugs or radiotherapy (Lang et al., 2019) can induce the production of excess ROS, leading to ferroptosis in lung cancer. Meanwhile, ferroptosis is involved in regulating the growth of tumors (Dolma et al., 2003), especially non-NSCLC. Since ferroptosis regulators are associated with chemotherapy resistance and immunotherapeutic effects on combating cancer, it is rational to make use of ferroptosis-related drugs to manage tumor therapy.

It was found that disrupting iron homeostasis made cells sensitized to ferroptosis in lung cancer (Kuang and Wang, 2019). Intracellular iron accumulation may accelerate lung tumorigenesis and development (Aleman et al., 2002). Reducing iron levels with DFX, and its analogs, inhibits tumor growth by limiting iron provision (Sarno et al., 2018). These results were favored by researchers who demonstrated that iron chelators can suppress the growth of human lung tumor xenografts (Lui et al., 2013). However, iron chelators were reportedly useless in treating patients with solid malignant tumors (Torti et al., 2018). In a study that enrolled 12 patients with advanced lung tumors, the results showed no significant difference between the groups using the iron chelator, triapine, with gemcitabine, or by using gemcitabine alone. As there was different efficacy of iron chelators in experimental and clinical trials, more studies are warranted.

Lung tissue is under higher oxidative stress than other tissues, and is characterized by increased expression of system Xc-. Blocking system Xc- using erastin, sorafenib (Li Y. et al., 2020), dihydroartemisinin (Yuan et al., 2020), long non-coding RNA NEAT1 (Wu and Liu, 2021), or sulforaphane (Iida et al., 2021; Table 1) to induce ferroptosis is a promising therapy for lung cancer. For example, erastin/sorafenib helped to sensitize the NSCLC cells to cisplatin through inhibition of the NRF2/xCT pathway (Li Y. et al., 2020), resulting in the suppression of tumor growth. Regarding NRF2, its activation was related to lung cancer development by triggering proliferation and angiogenesis (Lignitto et al., 2019). Inhibiting NRF2 promoted lipid peroxidation and triggered ferroptosis, which played a significant role in suppressing lung tumor development (Hammad et al., 2019). Recently, Gai et al. (2020) revealed that inhibiting NRF2 with the combination of erastin and acetaminophen (APAP) could increase the sensitivity of erastin-induced ferroptosis in NSCLC cells. Regarding GPX4, a study found that GPX4 activation in NSCLC cells and increased expression of GPX4 were related to a worse prognosis (Lai et al., 2019). Zhang et al. (2020) detected that higher expression of GPX4 was associated with a higher probability of drug resistance, but inhibiting GPX4 maintained the anti-tumor effect of cisplatin (Deng et al., 2021). Additionally, there were many other agents regulating ferroptosis through various mechanisms in lung cancer. For example, both zinc (Palmer et al., 2019), and celastrol (Liu et al., 2021) can induce ferroptosis by increasing the level of ROS. Additionally, Erianin (Chen P. et al., 2020) can trigger ferroptosis in a calcium/calmodulin-related manner. To summarize, the combination of conventional drugs and ferroptosis-related agents has a bright future in the treatment of lung cancer.

### Hepatocellular Carcinoma (HCC)

Hepatocellular carcinoma is a major cause of tumor-related death worldwide (Bray et al., 2018). Liver cancer has complicated pathological mechanisms, and can be mediated by a variety of factors. In early HCC, patients primarily benefit from surgical lesion resection and liver transplantation (Hsiao et al., 2020). However, due to the difficulty in diagnosing the condition early, as well as the rapid progression, most patients have either a locally advanced or a metastatic diagnosis, and may have missed the opportunity for surgery (Anwanwan et al., 2020). Therefore, the prognosis of HCC seems unsatisfactory.

Clinically, sorafenib and lenvatinib are the standard treatments for patients with advanced HCC, but their clinical efficacy is very limited due to drug resistance. Pharmacologically, sorafenib facilitates ferroptosis by inhibiting SCL7A11 to block the import of oxidized cysteine into the cell, ultimately functioning to kill HCC cells (Louandre et al., 2013). Enhancing the effect of sorafenib is worthy of our attention. Recently, Li et al. (2021) revealed that cysteine depletion made HCC cells more vulnerable to sorafenib-induced ferroptosis (Li et al., 2021), which provides us with a new perspective to enhance the effect of sorafenib. Research also showed that DFX can reduce the therapeutic effect of sorafenib in HCC cell lines (Saeki et al., 2016).

Several proteins or agents [e.g., DAZAP1 (Wang et al., 2021), MT-1G (Sun et al., 2021), BCAT2 (Wang et al., 2021), QSOX1 (Sun et al., 2021), and Solasonine (Jin et al., 2020), Table 1] exerted significant impacts on ferroptosis in HCC cells. Moreover, we also summarized relevant pathways worthy of being mentioned for their role in HCC-related ferroptosis. Sun et al. (2016) revealed the role of the p62-Keap1-NRF2 pathway in ferroptosis in HCC cells. Inhibiting the expression of NRF2 genetically or pharmacologically enhanced the anti-tumor effect of erastin and sorafenib by upregulating the levels of iron and ROS. Recently, Sun et al. (2021) found that QSOX1 could also inhibit NRF2 activation to promote sorafenib-induced ferroptosis. Besides, triggering the AMPK/SREBP1 pathway to inhibit the transcription of BCAT2 contributes to ferroptotic death in HCC (Wang et al., 2021).

Regarding proteins and their effect on promoting the growth of HCC, Bai et al. (2017) indicated that sorafenib and erastin can upregulate the expression of OS-related Sigma 1 receptor (S1R) protein, and S1R prevents HCC cells from undergoing ferroptosis. Obtaining and understanding the entirety of the roles of these proteins could provide us with several valuable biomarkers of HCC to forecast prognosis.

### Breast Cancer

Breast cancer is a malignant tumor responsible for substantial economic burden and mortality in women (Sullivan et al., 2011). Hormone therapy, chemotherapy, and targeted therapy are recommended. However, the prognosis of breast cancer has much room to improve. We are looking forward to discovering a promising way to control this malignancy. The relationship between anti-breast cancer drugs and ferroptosis is currently under investigation. Ferroptosis-inducing therapy for tumor treatment was a breakthrough discovery. It was observed that cells with resistance to the human lapatinib were sensitized to ferroptosis (Ma et al., 2017). Moreover, neratinib (Nagpal et al., 2019) and lapatinib induced ferroptosis in breast tumor cells by elevating the level of ROS (Ma et al., 2016). Women with triple-negative breast cancer (TNBC) had a poor prognosis, and a discovery showing that TNBC was sensitive to ferroptosis provided evidence for the potential of ferroptosis in TNBC treatment.

Some ferroptosis inducers [i.e., Sulfasalazine (Yu H.C. et al., 2019), Siramesine (Villalpando-Rodriguez et al., 2019), erastin@FA-Exo (Yu M. et al., 2019), and Curcumin (Li R.H. et al., 2020)] contribute to anti-cancer therapy in breast cancer. For instance, SAS can inhibit the system Xc- to enhance ferroptosis in various breast tumor cells, especially in cells with low estrogen receptor expression (Yu H.C. et al., 2019), exosome-based erastin (erastin@FA-Exo) can kill TNBC cells by promoting depletion of glutathione and excess production of ROS (Limoni et al., 2019). Wu et al. (2020) concluded that the overexpression of glycogen synthase kinase-3β enhanced the therapeutic effect of erastin-triggered ferroptosis in breast tumors. Besides, Zhang et al. (2021) found that circRHOT1-induced ferroptosis could be inhibited by the upregulation of STAT3 and miR-106a-5p in breast cancer. It is necessary to find more valuable targets and make rational use of targeted drugs to trigger ferroptosis, thereby enhancing the sensitivity of anti-tumor drugs in breast cancer.

### Pancreatic Cancer

Pancreatic ductal adenocarcinoma (PDAC) is commonly characterized by high mortality and risk of metastasis (Ferlay et al., 2018). Currently, surgery is an effective means of pancreatic cancer therapy. Gemcitabine has been widely used as the firstline therapeutic drug for the treatment of advanced PDAC. However, its 5-year survival rate is still low.

Dynamic regulation of iron metabolism and ROS metabolism is important in ferroptosis in the setting of PDAC. Non-oxidative dopamine and baicalein regulate those pathways, which are beneficial to pancreatic cancer therapy (Donald et al., 2012). Artesunate (ART) acts in an ROS- and an iron-dependent manner to induce ferroptosis and kill PDAC cells (Eling et al., 2015). Moreover, the combination of ART and GRP78 inhibitor worked in KRAS mutant PDAC (Wang et al., 2019). Ruscogenin can also induce ferroptosis by influencing the levels of ferroportin and transferrin to exhibit anticancer effects (Song et al., 2020). Yamaguchi et al. (2018) demonstrated triple combined treatment using Cotylenin A, Piperlongumine, and sulfasalazine as a successful therapeutic method against PDAC. Recently, Badgley et al. (2020) proved that depletion of cysteine triggered ferroptosis in pancreatic tumors in mice, suggesting that an agent exhausting cysteine to induce ferroptosis in PDAC may be a promising therapy.

There are genes that play roles in pancreatic cancer by regulating ferroptosis. ADP Ribosylation Factor 6 is a part of the Ras family, and can endow pancreatic cancer cells to be sensitive to OS, especially RSL3-induced lipid peroxidation (Ye Z. et al., 2020). Moreover, ARF6 can regulate gemcitabine resistance to exhibit a profound effect on PDAC development (Ye Z. et al., 2020). Nuclear receptor coactivator 4 (NCOA4)-regulated ferritinophagy reportedly enhanced ferroptosis through the degradation of ferritin and ROS generation in PDAC. Knockdown of NCOA4 in PDAC restrained ferritinophagy and inhibited erastin-triggered ferroptosis, but the NCOA4 overexpression could increase the degradation of ferritin and trigger ferroptosis (Hou et al., 2016). HSPA5 (heat shock protein family A member 5) acts on GPX4 to negatively regulate ferroptosis in pancreatic cancer. There was a study suggesting that HSPA5 can overcome gemcitabine resistance by triggering ferroptosis. In short, targeting ferroptosis is a promising strategy in PDAC therapy (Zhu et al., 2017).

## Ferroptosis-Associated Anti-Cancer Drugs in Preclinical Experiments and Clinical Trials

The concentration of iron, the accumulation of ROS, and the accumulation of lipid peroxidation simultaneously affect ferroptosis in tumor cells, providing the opportunity for targeted therapy (Dixon et al., 2012). To date, there is emerging evidence confirming the effect of ferroptosis induction for tumor therapy in experimental models, especially for advanced malignant tumors that are frequently resistant to common therapies (Figure 1). However, there is no clinical trial with ferroptosis-associated agents showing substantial tumor shrinkage or prolongation in progression-free survival. Herein, we list the ferroptosis-associated antitumor drugs (Table 2), hoping to provide direction for further translational research.

**TABLE 2:** Summary of ferroptosis-associated anti-tumor agents.

**Ferroptosis-associated antitumor agents**	**Mechanism**	**Associated Cancer types**
Erastin and analogs	Inhibit system xc-; alter the permeability of VDAC; activate p53	Diffuse large B cell lymphoma (Yang et al., 2014); lung cancer (Yamaguchi et al., 2013); rhabdomyosarcoma cells (Codenotti et al., 2018); glioblastoma (Chen et al., 2015); head and neck cancer (Lee et al., 2020); ovarian cancer cells; acute myeloid leukemia cells (Yu et al., 2015)
Sulfasalazine	Inhibit system xc-	Breast cancer cells (Yu H.C. et al., 2019); head and neck cancer (Kim et al., 2018)
Sorafenib	Inhibit system xc-	Hepatocellular Carcinoma (Louandre et al., 2015; Sun et al., 2016)
(1S, 3R)-RSL3	Inhibit GPX4 directly	Head and neck cancer (Shin et al., 2018)
FIN56	Degrade GPX4; bind to SQS; deplete antioxidant CoQ10	HT-1080 fibrosarcoma cells (Shimada et al., 2016)
FINO2	Oxidize ferrous iron; inactivate GPX4 indirectly	HT-1080 fibrosarcoma cells (Gaschler et al., 2018)
Artesunate	Induction of reactive oxygen species (ROS); mitochondrial impairments and SLC7A11-involved glutathione depletion	Ovarian cancer cells (Greenshields et al., 2017; Roh et al., 2017); Huh7 cell, pancreas cancer (Roh et al., 2017), Adult T-cell leukemia/lymphoma (Ishikawa et al., 2020)
Ruscogenin	Increase the concentration of intracellular ferrous irons and the production of ROS	Pancreas cancer (Song et al., 2020)
H_2_O_2_/Fe_3_O_4_-PLGA nanosystem	Inhibit system xc-; Fenton reaction	4T1, HT-1080, Hep G2, CT26 (Li et al., 2016)
PEGylated single-atom Fe-containing nanocatalysts (PSAF NCs)	Fenton reaction	4T1 tumor cells (Huo et al., 2019)
Low-Density Lipoprotein nanoparticles	GSH depletion	Hepatocellular Carcinoma (Ou et al., 2017)

### Erastin and Analogs

Erastin is one of the most thoroughly studied agents of ferroptosis induction. It not only induces non-apoptotic iron-dependent death, but sensitizes cancer cells to chemotherapeutic and radiotherapeutic treatments. As imidazole ketone erastin (IKE) and piperazine erastin (PE) have poor solubility in water and relative lability, the solubility should be taken in consideration when drugs were designed. Erastin analogs are capable of inhibiting tumor growth, and they have minimal toxicity both *in vitro* and *in vivo*. One study revealed that in a diffuse large B cell lymphoma mouse model, IKE can exert an anti-tumor effect with no side effects (Zhang et al., 2019). In addition to inducing ferroptosis, erastin, when combined with chemotherapeutic drugs, can enhance the chemosensitivity of cancer cells. Experimental results suggest that erastin can increase lung cancer cell sensitivity to cisplatin (Yamaguchi et al., 2013), can increase rhabdomyosarcoma cell sensitivity to actinomycin D (Codenotti et al., 2018), and can increase glioblastoma cell sensitivity to temozolomide (Chen et al., 2015). Moreover, erastin reportedly diminished the drug resistance of various kinds of chemotherapy-resistant cells, such as ovarian cancer cells and acute myeloid leukemia cells (Yu et al., 2015; Sato et al., 2018; Lee et al., 2020).

In addition to the function of inducting ferroptosis and enhancing sensitivity to chemotherapy, erastin could make tumor cells more sensitive to radiation. The goal of radiotherapy is to ensure the anti-tumor effect without increasing the toxicity and side effects of normal tissues. The combined therapy of erastin and radiation can meet our expectations. In a mouse model of sarcoma, IKE, when combined with stereotactic radiation, had synergistic effects on tumor volume shrinkage (Ye L.F. et al., 2020). In a cell model of lung tumor, the combination of erastin and radiation in the co-treated group reportedly induced significant inhibition of tumor growth (Ye L.F. et al., 2020).

### Sulfasalazine (SAS)

Sulfasalazine is an FDA-approved agent medically used for rheumatoid arthritis. SAS can trigger ferroptosis in breast tumor cells, particularly in cells with lower expression of ER (Yu H.C. et al., 2019). In the era of personalized medicine, this study could be provided as a reference for decision-making for selective patients. Although SAS could induce ferroptosis, cancer cells can develop relative mechanisms to escape cell death. However, a recent study reported that silencing the CDGSH iron-sulfur domain-containing protein 2 gene rendered resistant head and neck cancer cells susceptible to sulfasalazine-triggered ferroptosis. Pioglitazone sensitized resistant HNC cells to sulfasalazine treatment, and caused over-accumulation of iron and ROS (Kim et al., 2018).

### Sorafenib

Sorafenib is also a clinical multi-kinase inhibitor, which is widely applied in the treatment of various advanced carcinoma. NRF2 and Rb, as tumor suppressor genes, can suppress sorafenib-triggered ferroptosis specifically in hepatocellular carcinoma cells. The expression of Rb and NRF2 is a vital factor in determining the therapeutic effect of ferroptosis-related therapies in HCC cells. Therefore, pharmacologic or genetic inhibition of NRF2 and Rb expression or activity in hepatocellular carcinoma cells BAL b/c nude mouse xenograft model treated with sorafenib could enhance the effects of ferroptosis (Louandre et al., 2015; Sun et al., 2016). Based on the above results, Rb and NRF2 could be a checkpoint for ferroptosis-associated therapy in clinical practice.

### (1S, 3R)-RSL3

RSL3 functions by regulating GPX4 to render lipid peroxidation and ferroptosis induction. Reports have illustrated that (1S, 3R)-RSL3 shows lethality toward the BJ-derived cell system (Liang et al., 2019). GPX4 inhibition could render therapy-resistant tumor cells more sensitive to ferroptosis, but a variety of tumor cells can evade RSL3-induced ferroptosis. Fortunately, recent studies have suggested that activating the p62 and NRF2-ARE pathways could cause the resistance of HNC cells to GPX4 inhibition. Meanwhile, the inhibition of it could reverse ferroptosis resistance in a mouse model transplanted with HN3R (Shin et al., 2018).

### FINO2

FINO2 could inhibit GPX4 indirectly and oxidize iron directly, ultimately resulting in lipid peroxidation (Gaschler et al., 2018), which initiates a dual mechanism of ferroptosis. Thus far, there are not any *in vivo* or *in vitro* trials evaluating whether FINO2 is of high specificity and presenting low side effects in both preclinical and clinical settings.

### FIN56

FIN56, which is derived from CIL56, is a known inducer of ferroptosis. FIN56 is more specific than CIL56 in triggering ferroptosis. There are two pathways that distinctly contribute to ferroptosis by FIN56. Firstly, FIN56 regulates GPX4 degradation with the help of enzymatic acetyl-CoA carboxylase. Secondly, FIN56 specifically activates the enzyme squalene synthase, causing intracellular antioxidant CoQ10 to deplete, which would increase tumor cell sensitivity to FIN56-triggered ferroptosis. Moreover, idebenone is proven to be the only inhibitor of FIN56-induced ferroptosis (Shimada et al., 2016).

### Artesunate

Artesunate, a type of traditional Chinese medicine, could inhibit the growth of ovarian cancer cells, as well as decrease cancer cell growth in a mouse model of ovarian malignancy. It could strongly induce the production of ROS. It works in a dose-dependent manner. Exposure of ovarian cancer cells to high concentrations of Artesunate caused ROS-related ferroptosis (Greenshields et al., 2017; Roh et al., 2017). Meanwhile, Nrf2 inhibition could reverse the resistance of head and neck tumor cells to ART-induced ferroptosis (Roh et al., 2017). Besides, ART synergizes with sorafenib to trigger ferroptosis in hepatocellular carcinoma (Li Z.J. et al., 2020). Sorafenib blocks GSH synthesis directly, whereas ART promotes the activation of the lysosome. Combining ART with sorafenib would promote cathepsin B/L activities, eventually resulting in ferroptosis. Recent studies have suggested that ART also showed antitumor activity in a mouse Adult T-cell leukemia/lymphoma model (Ishikawa et al., 2020).

### Ruscogenin

Ruscogenin, another form of traditional Chinese medicine, was found to have anticancer activity. It functions by enhancing the production concentration of ferrous irons and the concentration of ROS. In the nude mouse xenograft model, ruscogenin impaired the viability of PDAC cell-induced death. Moreover, it is time- and dose-dependent. Furthermore, in BxPC-3 human pancreatic cancer xenografts, there are significant antitumor effects without any toxicity detected in normal tissues (Song et al., 2020).

### Nanoparticle Inducers

Because current inducers of ferroptosis have poor biodegradability and biocompatibility, there remains a gap between the current results and our expectations. Ferroptosis-driven nanoparticle inducers, with improved therapeutic effects and reduced side effects, would narrow the gap.

Extensive research has shown that the iron-dependent chemical reaction, known as the Fenton reaction, has high efficacy in combating tumors by inducing ferroptosis (Qian et al., 2019). A variety of newly developed nanotherapeutics can trigger the Fenton reaction in tumor cells (Qian et al., 2019). Recently a high-performance catalytic nanosystem, the iron engineered framework of mesoporous silica nanoparticles (MSNs), was developed for efficient cancer therapy. The report showed that in *in vivo* experiments, MSNs could induce ferroptosis and apoptosis simultaneously and without toxicity (Huo et al., 2019). The H_2_O_2_/Fe_3_O_4_-PLGA nanosystem, triggered by ultrasound treatment (Li et al., 2016) and PEGylated single-atom Fe-containing nanocatalysts (PSAF NCs) (Huo et al., 2019), could facilitate the tumor-localized Fenton reaction. Tumor growth curves showed a downward trend with different treatments compared with the controlled group.

Nanoparticle-induced ferroptosis is also mediated by the inactivation of GPX4. Sorafenib is not only a GPX4 inhibitor, but also an encapsulated nanostructure that comprises Fe^3+^ iron and tannic acid. Tannic acid (TA) could supply sustainable Fe^2+^ to maintain tumor-localized Fenton reaction (Liu et al., 2018). Therefore, nanoparticle inducers (combining sorafenib with TA) could induce ferroptosis in dual pathways. Moreover, low-density lipoprotein nanoparticles, reconstituted with docosahexaenoic acid (DHA), were proven to induce ferroptosis by inhibiting the activity and expression of GPX4 in rat and human hepatic cancer cell lines (Ou et al., 2017).

## Hypothesis

Currently, the therapeutic effects of traditional therapy fail to meet clinical satisfaction due to intrinsic or acquired drug resistance. When the refractory or relapsed tumor becomes resistant to current antitumor therapy, we should consider ferroptosis-associated therapy. Since the relationship between ferroptosis and correlated drug discovery has been gradually revealed, a growing number of small molecules have shown the ability to trigger ferroptosis either directly or indirectly. Ferroptosis is mediated by the metabolism of iron and lipids, which is one component of the normal physiological process. Besides, ferroptosis is associated with many biological processes, including NADPH, fatty acid metabolism, and regulation of glutathione level (Stockwell et al., 2017). When Inducing ferroptosis, it may result in imbalanced homeostasis. Therefore, the targets or hallmarks of ferroptosis-associated therapy, such as the gene or protein expressed, must be studied to maximize specificity, minimize unpleasant side effects, and determine which tumor types have a tendency to be the target of ferroptosis induction.

Above all, we suggest a hypothesis that ferroptosis plays a vital role in tumor development with a dynamic balance of iron and lipid levels, and that ferroptosis-associated therapy can have anti-tumor effects, especially when combined with chemotherapy and radiotherapy.

## Discussion and Perspectives

This review describes the underlying mechanisms of ferroptosis and summarizes its role and potential therapy in solid tumors. In short, ferroptosis restrained tumor growth and compensated for the unsatisfactory effects of current treatments (Dixon et al., 2012). These ferroptosis-associated anti-tumor agents can be utilized in tumors throughout the body. The effect of ferroptosis induction is more efficient and rapid, the effect lasts longer, and very minimal concentration is needed. However, ferroptosis is related to other diseases (Tang et al., 2018; Alim et al., 2019; Fang et al., 2019). Consequently, the specificity and minimal side effects of ferroptosis regulators in preclinical and clinical settings warrant further elucidation.

To date, research has shown a link between ferroptosis and ferroptosis-related drug discovery. Many small molecules have been confirmed to trigger ferroptosis by regulating iron and lipid peroxidation. More importantly, ferroptosis inducers can enhance the chemosensitivity of many kinds of cancer cells and enhance the radiosensitivity of cancer cells. Therefore, they may have potential clinical application as a novel chemotherapy drug or radiosensitizer. However, ferroptosis induction has dual roles in tumor growth. Some drugs induce ferroptosis to slow down tumor growth, but ferroptosis itself could evoke immunosuppression to hasten tumorigenesis. The mechanisms warrant illumination, and combining ferroptosis-inhibitors with immunotherapy may be a novel strategy. Meanwhile, considering that iron deficiency and iron overload may have an impact on anti-tumor activity, studies are needed to uncover the suitable iron concentration and optimal dose of ferroptosis-related drugs to minimize tumor progression. Lastly, some ferroptosis inducers could mediate a single pathway, but others can mediate various pathways. Thorough studies are necessary to unveil effective multimodality therapies.

## Author Contributions

HW, DL, and QY wrote the manuscript. CL, AS, YD, and QW supervised the research, led the discussion, and revised the manuscript. All authors analyzed and discussed the literature, commented the manuscript, and read and approved the final manuscript.

## Conflict of Interest

The authors declare that the research was conducted in the absence of any commercial or financial relationships that could be construed as a potential conflict of interest.
